# Effect of laser heat treatment on Pull-out bond strength of fiber posts treated with different silanes

**DOI:** 10.4317/jced.54145

**Published:** 2018-05-01

**Authors:** Fereshteh Shafiei, Maryam Saadat, Zahra Jowkar

**Affiliations:** 1 Professor, Oral and Dental Disease Research Center, Department of Operative Dentistry, School of Dentistry, Shiraz University of Medical Sciences, Shiraz, Iran; 2 Assistant professor, Department of Operative Dentistry, School of Dentistry, Shiraz University of Medical Sciences, Shiraz, Iran

## Abstract

**Background:**

This study evaluated the effect of three different silanes and post-silanization treatments on the retentive strength of fiber posts luted with an etch-and-rinse resin cement.

**Material and Methods:**

One hundred intact maxillary central incisors were randomly divided into 10 groups after endodontic treatment and post space preparation (n=10). The fiber posts were etched using 24% hydrogen peroxide. Posts of the control group did not receive silane. In nine experimental groups, each of the three silanes used, Scotchbond Universal adhesive, Bis-Silane and Porcelain Primer, was subjected to three treatments: air-drying at 25°C, warm air-drying and CO2 laser heat treatment. After cementation of the treated posts using One-Step Plus/Duo-Link cement, the specimens were stored for one weak and then subjected to pull-out bond strength (PBS) testing. The data in Newton (N) were analyzed using two-way ANOVA and Tukey tests (α=0.05).

**Results:**

PBS was significantly affected by silane type and post-silanization treatment (*p*<0.001). The interaction of the two factors was not statistically significant (*p*=0.15). The effect of Porcelain Primer on PBS was significantly higher than those of universal adhesive (*p*<0.001) and Bis-Silane (*p*=0.01), with similar results for the two latter. Warm air-drying and laser treatment significantly increased PBS (*p*<0.001). The lowest and highest PBS was obtained in the control (no silane) group (190.9±31) and laser-treated/ Porcelain Primer group (377.1±50), respectively.

**Conclusions:**

Warm air-drying and CO2 laser heat treatment had a significantly beneficial effect on retentive strength of fiber posts. Porcelain Primer was significantly more effective than universal adhesive and Bis-Silane.

** Key words:**Laser heat treatment, Pull-out bond strength, fiber post.

## Introduction

Use of fiber-reinforced composite post (FP) has increased to provide retention for the final restoration in endodotically treated teeth (1). This is due to esthetic advantages, elastic modulus similar to that of dentin and bonding ability to root canal dentin (2). In the adhesively cemented post, a bonded unit among post, adhesive system/resin cement and root dentin is achieved through adequately strong bond at the post‒cement and cement‒dentin interfaces (2). Weak bonding at each interface could result in failure of the post-retained restoration (3). The presence of a relatively smooth surface along with highly polymerized epoxy resin covering of FPs prevents any mechanical interlocking or chemical interaction with resin cements (2,4). Therefore, various surface pretreatment methods have been investigated to overcome the unreactivity of the FRC post surface (3,4).

Organosilanes, with an intrinsic dual reactivity, act as an adhesion promoter between the inorganic surface and polymeric resins through an organic functional group and three alkoxy groups (2,5). FPs can well benefit from the silanization for resin bonding if their glass fibers are exposed and available for formation of siloxane bonds with silane (2-4). This could be provided by appropriate surface pretreatment (6). Hydrogen peroxide (H2O2) is capable of partially dissolving the epoxy resin and breaking epoxy resin bonds (4). A 1-minute application of 24% and 50% H2O2 exposed glass fibers for chemical bonding and provided spaces for penetration of the resin adhesive (7), increasing bond strength of the resin to the silane-treated undamaged FPs (3,4). Nevertheless, silanization is found to be a technique-sensitive step and its efficacy is affected by compositional variables (pH, solvent content, molecule size), hydrolysis time, application method and drying procedure (4,8,9). Heat treatment subsequently to silanization was demonstrated to be beneficial for improving the effectiveness of silane treatment (10). This approach was implemented using warm air drying, possibly facilitating solvent evaporation (10,11). Although solvent content may be beneficial to enhancing silane wetting, incomplete elimination may compromise coupling (4).

Carbon dioxide (CO2) laser is one of the most commonly used lasers in dental practice. The beneficial effect of CO2 laser beams on the performance of silane coupling agent and on improving bonding of resin to ceramic was reported by Chen *et al.*. This effect was attributed to surface warming induced by a focused CO2 laser beam at low energy setting (12).

Recently, a new category of adhesive systems was introduced as universal adhesives. In Scotchbond Universal, methacryloxydecyl phosphate monomer and silane coupling agent are combined, enabling bonding to different substrates such as silica-based ceramics (13). It might be used in lieu of silane on FPs surface due to the bonding ability to the exposed glass fibers.

Therefore, this study was designed to test the null hypothesis that CO2 laser treatment, compared to air- and warm air-drying and different silane types, has no effect on the retentive strength of FP cemented in the root canal.

## Material and Methods

One hundred human maxillary central incisors with similar size and anatomic shape and straight roots without cracks, were selected and stored in 0.5% chloramines-T solution at 4°C until use. They were used following informed consent from patients and approval of the research protocol by the local Ethics Committee. The roots were sectioned to provide a uniform length of 12 mm, using a water-cooled diamond saw (D&Z, Berlin, Germany). They were embedded in epoxy resin blocks in the vertical axis and were endodontically treated.

The specimens were stored in water for one week for the complete setting. Afterwards, a 5-mm-deep post space was prepared using a #2 drill from the respective post manufacturer by the same operator. Cleanliness of the root walls was confirmed by radiographs. Glass fiber posts (TransLuma Post ISO #100, Bisco, Schaumburg, IL, USA) were tried in the post space for a passive fit in the prepared depth without a need for relining. Post surfaces were etched using 24% hydrogen peroxide for one minute, water-rinsed and air-dried.

The FPs were divided into one control group and nine experimental groups (n=10), based on three silane types and three post-silanization treatments. No silane was applied in the control group. In groups 2-4, Scotchbond Universal adhesive (3M ESPE) was applied onto post surface for 20 sec and then air-thinned/dried for 10 sec at 25°C from a distance of 10 mm in the air-dried group. In the warm air-dried group, air-thinning/drying was performed at 50 ± 5°C using a blow dryer (Gordak 952, Guangdong, China, nozzle size of 3mm). In the laser-treated group, following air-thinning/drying, the post surface was subjected to the CO2 laser (DS-40U/Daeshin Enterprise, Seoul, Korea, a wavelength of 10.6 µm) heat treatment at 1 W in continuous mode from a distance of 30mm for 20 sec with a size tip diameter of 1 mm. In a preliminary study, the irradiation parameters used were tested to confirm the temperature produced (50 ± 5°C) on the post surface. In the three groups, the temperature used was checked by a laboratory thermometer (KEW 1011, Kyoritsu, Tokyo, Japan) by placing the electrode on the post surface. Finally, the adhesive was cured for 10 sec with a light-curing unit (VIP Junior, Bisco) at 600 mW/cm2.

In groups 5-7, two-bottle silane solution Bis-Silane (Bisco) was mixed and applied on the post surface according to manufacturer’s instructions.

In groups 8-10, one-bottle silane Porcelain Primer (Bisco) was applied in two layers according to manufacturer’s instructions.

Etch-and-rinse resin cement One-Step Plus/Duo-Link Universal (Bisco) was used to cement the treated posts. After acid etching and water rinsing, the root canals were blot-dried with paper points. Then One-Step Plus was applied according to manufacturer’s instructions. The mixed cement was applied into the post space and onto the post surface. The post was immediately seated with a slight vibratory motion and held under finger pressure for 10 sec. The excess cement was removed and light polymerization was carried out for 60 sec using VIP Junior light-curing unit at 600mW/cm2. The specimens were stored in distilled water at 37°C for one week.

For pull-out testing, a tensile force was applied parallel to the long axis of both the post and root at a crosshead speed of 1mm/min in a universal testing machine (Zwick, Roell, Ulm, Germany) until the post was dislodged. At that moment, the maximum load was recorded in Newton (N). Data in all the groups were analyzed with one-way ANOVA and post-hoc Tukey tests. Ignoring the control group, two-way ANOVA was used to analyze the effect of two main factors (different silanes and treatments). Multiple comparisons were performed with Tukey tests (α=0.05).

The dislodged posts were examined under a stereomicroscope (Carl Zeiss Inc, Oberkochen, Germany) at ×20 to determine the failure modes as follows: adhesive failure at the cement‒dentin interface, adhesive failure at the cement‒post interface, mixed failure as a combination of adhesive failures along with cohesive failure within the resin cement (the separated cement). The representative specimens of the dislodged posts were observed under a scanning electron microscope (SEM, Tescan, Vega II, England).

## Results

Pull-out bond strength (PBS) mean values and standard deviations (in N) for the nine study groups are presented in  Table 1.

**Table 1:**
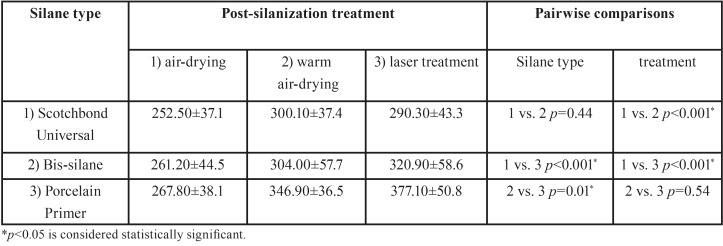
Mean pull-out bond strength (N) and standard deviation (SD) for nine experimental groups.

Considering the control group, the results of one-way ANOVA and post hoc Turkey tests revealed a significantly lower PBS in the control group (no silane) compared to those in the nine other groups (*p*≤0.01).

Two-way ANOVA indicated that PBS was influenced by silane type and post-silanization procedures (*p*<0.001). The interaction between the two factors was not significant (p=0.15). The effect of air-drying was significantly lower than that of warm air-drying and laser treatment (*p*<0.001); the two latter did not differ (*p*>0.05). The effect of Porcelain Primer was significantly higher compared to those of universal adhesive (*p*<0.001) and Bis-Silane (*p*=0.01), with a similar result for the two latter.

**Figure 1:**
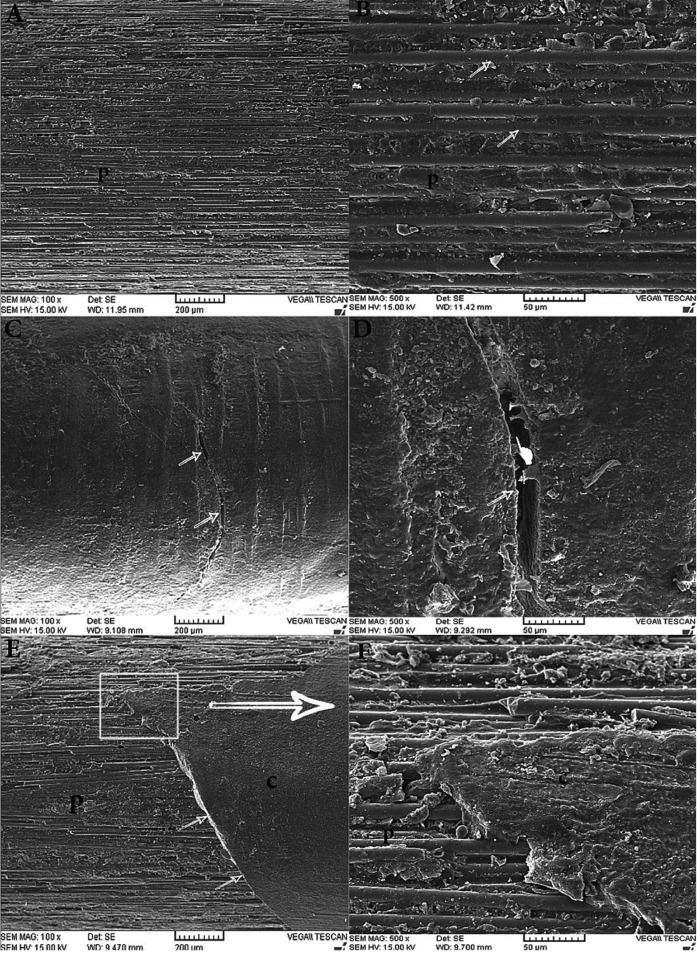
Representative SEM images of the failure modes of the dislodged posts after pull-out testing at magnifications of ×100 and ×500. A and B, adhesive failure at the cement‒post interface, showing the exposed post surface free of the cement; arrow, exposed glass fibers. C and D, adhesive failure at the cement‒dentin interface, showing the post surface that is completely covered with cement; arrow, deep crack in the cement; pointer, exposed fiber at the depth of the crack. E and F, mixed failure, showing part of the exposed post surface along with a part of cement covering; arrow, fracture line of the cement.

The distributions of failures are described in  Table 2. The failure modes for the control group and air-dried groups were mainly adhesive at the cement‒post interface or mixed failure so that the dislodged posts were free of the cement or with some parts of the cement on the post surface. In the laser- and warm air-treated groups, all the posts were completely or partially covered with the cement, indicating adhesive failure at the cement‒dentin interface or mixed failure. Representative SEM images of the failure modes of the dislodged posts are presented at two magnifications of ×100 and ×500 in Fig 1.

**Table 2:**
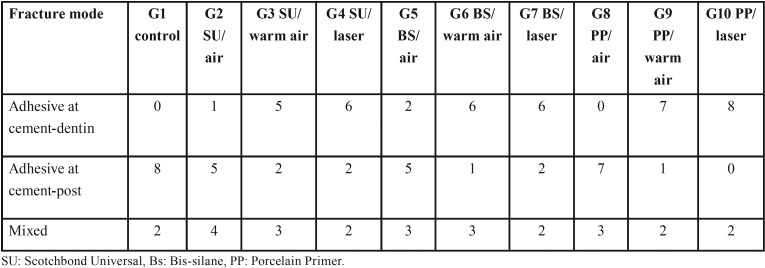
Mode of fracture of the dislodged posts after pull-out testing for the ten groups.

## Discussion

In the present study, the effects of various post-silanization procedures on the retentive force of differently silanized FRC posts in the root canal were assessed using pull-out tests. When comparing this test with the push-out test, the pull-out test evaluates total debonding resistance of the complete bonding surface of the FP in the entire length of the root canal (14), allowing simultaneous evaluation of the shear and tensile stresses involved with more clinical relevance (15). The post length of 7-10 mm is commonly used for in vitro testing and in the clinic. However, some authors reported that the high retentive forces provided by adhesive cement in this post length resulted in root fracture before post debonding (15). Our preliminary tests with 8-mm post length led to breakage of post extensions prior to post debonding for some specimens similar to that reported by Soejima *et al.* (14). Therefore, a 5-mm post length was selected. A recent systematic review on the efficacy of post silanization concluded that when FRC posts were cemented into the natural root canal, the combination of appropriate post pretreatment for exposure of the glass fibers plus silanization significantly improved the post retention.5 In this study; all the silanized post groups yielded higher retentive strength compared to no silane-treated group, regardless of post-silanization procedure. 24% H2O2 pretreatment of the posts was carried out prior to silanization in all the groups1`, providing a reactive surface to silanization (7). In all the tested groups, the same adhesive resin cement was used for post cementation, allowing us to focus on the variables related to silanes and post-silanization treatments. The obtained results indicated that the heat treatments improved the retentive strength. In addition, Porcelain Primer performed significantly better than universal adhesive and Bis-Silane. Thus the null hypothesis can be rejected. Post-silanization treatment (such as warm air-drying) is commonly performed to improve the bond strength of composite to ceramic through accelerating the chemical interaction mechanism between them (10). Elimination of water, alcohol and volatile by-products during completion of the silane-silica condensation and the resultant facilitation of covalent bond formation are responsible for this effect. Two-component silane was reported to be more sensitive to heating (16,17). A similar approach has been proposed to improve bonding to silanized post. Firstly, Monticelli *et al.* indicated that air drying at 38°C is able to increase the microtensile bond strength of resin to hydrogen peroxide-treated post (18). When a silane is applied to the post surface and then dried, a thick interphase layer is formed, which might be considered as the weak link of the bond with a lubrication effect (9). Warm air-drying may facilitate elimination of the outermost layers consisting of small oligomers physisorbed to the innermost layer. The innermost layer is the only required cross-linked layer, providing a strong siloxane bond (10,18).

Consistent with our results, the beneficial effect of heat drying of the silanized post at 60°C on bonding to the resin cement was also confirmed in a recent study (19). However, this effect has not been previously reported for non-treated FP at 38°C with a two-bottle silane (20) and at 60°C with the different one- and two-bottle silane agents (21). In the above-mentioned studies, the differences in the results were attributed to different silane compositions and solvent contents. Comparison of our finding with other conflicting results may indicate the important role of etching prior to silanization. Furthermore, the present study is the only study to evaluate the effect of silane heat treatments on the pull-out bond strength of fiber posts in natural root canals. Similarly, Samimi *et al.* reported that silane heat treatment, including warm water and warm air on etched posts, the increased push-out bond strength in root dentin slices (22). The beneficial effect of warm air stream on solvent evaporation of simplified adhesives has been demonstrated. This resulted in the formation of high cross-linked polymers at adhesive‒dentin interface (23), contributing to the higher PBS in warm-air and laser heat-treated groups of the universal adhesive.

The other finding in this study was that CO2 laser treatment was as effective as warm air-drying in the bonding performance of fiber silanization with the resin cement. In Raman spectroscopy and bond strength test study, increased bond between the resin and porcelain was associated with a decrease in isolated Si-OH groups that was indicated following CO2 laser treatment of silane-treated porcelain (12).

A universal adhesive system containing silane, i.e. Scotchbond Universal, might be suggested to summarize silane and adhesive resin steps in one step (13). In the current study, the bonding performance of this new adhesive was similar to that of Bis-Silane; however, Porcelain Primer was significantly the better than the two others. Silane agent, water, ethanol, acidic monomer and Bis-GMA exist in the one-bottle universal adhesive. The interfering effect of Bis-GMA or acidic monomers on chemical interaction of the silane with silica-based porcelain, compared to pure silane, has been recently reported (24).

The better performance of prehydrolized one-bottle silanes was related to a greater number of available silanol groups compared to two-bottle silane in which hydrolysis occurs after mixing silane and hydrolysis-activating acid in two separate bottles (8,25). However, others reported that application of this freshly formed active silane might be more effective (16,26).

Furthermore, contrary to ethanol contained in Bis-Silane, one-bottle Porcelain Primer used in this study contains an acetone solvent. Previously, better bond strength of the acetone-based adhesive was reported due to the ability of acetone for slight dissolution of epoxy resin, roughening the post surface (27).

In evaluation of the failure modes, the non-treated group and air-dried groups for the three silanes used exhibited adhesive failure at cement‒post interface or mixed as the main failure mode. Strengthening cement‒post interface using heat treatment led to shifting of the failure mode to the cement‒dentin interface as a weak link. All the groups tested exhibited mean pull-out bond strength values higher than 200 N, as the minimum tensile bond strength for clinical success (28). Following post cementation, the final restoration was placed using composite resin or crown and subjected to complex forces from different directions. Therefore, further studies involving long-term mechanical and thermal cycling are necessary to confirm these results.

Within the limitations of the present study, the following could be concluded:

1) Porcelain Primer was significantly more effective in enhancing retentive strength of the posts cemented in root canals than Bis-Silane and one-bottle universal adhesive containing silane.

2) Warm air-drying of silanized post considerably improved the efficacy of the silanes tested. Alternatively CO2 laser treatment might be effective when a blow drier is not accessible in the dental clinic.

## References

[1] Naumann M, Koelpin M, Beuer F, Meyer-Lueckel H (2012). 10-year survival evaluation for glass-fiber-supported postendodontic restoration: a prospective observational clinical study. J Endod.

[2] Bitter K, Kielbassa AM (2007). Post-endodontic restorations with adhesively luted fiber-reinforced composite post systems: a review. Am J Dent.

[3] Yenisey M, Kulunk S (2008). Effects of chemical surface treatments of quartz and glass fiber posts on the retention of a composite resin. J Prosthet Dent.

[4] Monticelli F, Osorio R, Sadek FT, Radovic I, Toledano M, Ferrari M (2008). Surface treatments for improving bond strength to prefabricated fiber posts: a literature review. Oper Dent.

[5] Lung CY, Matinlinna JP (2012). Aspects of silane coupling agents and surface conditioning in dentistry: an overview. Dent Mater.

[6] Moraes AP, Sarkis-Onofre R, Moraes RR, Cenci MS, Soares CJ, Pereira-Cenci T (2015). Can Silanization Increase the Retention of Glass-fiber posts? A Systematic Review and Meta-analysis of In Vitro Studies. Oper Dent.

[7] de Sousa Menezes M, Queiroz EC, Soares PV, Faria-e-Silva AL, Soares CJ, Martins LR (2011). Fiber post etching with hydrogen peroxide: effect of concentration and application time. J Endod.

[8] Filho AM, Vieira LC, Araujo E, Monteiro Junior S (2004). Effect of different ceramic surface treatments on resin microtensile bond strength. J Prosthodont.

[9] Liu Q, Ding J, Chambers DE, Debnath S, Wunder SL, Baran GR (2001). Filler-coupling agent-matrix interactions in silica/polymethylmethacrylate composites. J Biomed Mater Res.

[10] Shen C, Oh WS, Williams JR (2004). Effect of post-silanization drying on the bond strength of composite to ceramic. J Prosthet Dent.

[11] Fabianelli A, Pollington S, Papacchini F, Goracci C, Cantoro A, Ferrari M (2010). The effect of different surface treatments on bond strength between leucite reinforced feldspathic ceramic and composite resin. J Dent.

[12] Chen JR, Oka K, Kawano T, Goto T, Ichikawa T (2010). Carbon dioxide laser application enhances the effect of silane primer on the shear bond strength between porcelain and composite resin. Dent Mater J.

[13] Alex G (2015). Universal adhesives: the next evolution in adhesive dentistry?. Compend Contin Educ Dent.

[14] Soejima H, Takemoto S, Hattori M, Yoshinari M, Kawada E, Oda Y (2013). Effect of adhesive system on retention in posts comprising fiber post and core resin. Dent Mater J.

[15] Tian Y, Mu Y, Setzer FC, Lu H, Qu T, Yu Q (2012). Failure of fiber posts after cementation with different adhesives with or without silanization investigated by pullout tests and scanning electron microscopy. J Endod.

[16] Barghi N, Berry T, Chung K (2000). Effects of timing and heat treatment of silanated porcelain on the bond strength. J Oral Rehabil.

[17] Hooshmand T, van Noort R, Keshvad A (2002). Bond durability of the resin-bonded and silane treated ceramic surface. Dent Mater.

[18] Monticelli F, Toledano M, Osorio R, Ferrari M (2006). Effect of temperature on the silane coupling agents when bonding core resin to quartz fiber posts. Dent Mater.

[19] de Rosatto CM, Roscoe MG, Novais VR, Menezes Mde S, Soares CJ (2014). Effect of silane type and air-drying temperature on bonding fiber post to composite core and resin cement. Braz Dent J.

[20] Kim HD, Lee JH, Ahn KM, Kim HS, Cha HS (2013). Effect of silane activation on shear bond strength of fiber-reinforced composite post to resin cement. J Adv Prosthodont.

[21] Novais VR, Simamotos Junior PC, Rontani RM, Correr-Sobrinho L, Soares CJ (2012). Bond strength between fiber posts and composite resin core: influence of temperature on silane coupling agents. Braz Dent J.

[22] Samimi P, Mortazavi V, Salamat F (2014). Effects of heat treating silane and different etching techniques on glass fiber post push-out bond strength. Oper Dent.

[23] Reis A, Carrilho M, Breschi L, Loguercio AD (2013). Overview of clinical alternatives to minimize the degradation of the resin-dentin bonds. Oper Dent.

[24] Chen L, Shen H, Suh BI (2013). Effect of incorporating BisGMA resin on the bonding properties of silane and zirconia primers. J Prosthet Dent.

[25] Anagnostopoulos T, Eliades G, Palaghias G (1993). Composition, reactivity and surface interactions of three dental silane primers. Dent Mater.

[26] Foxton RM, Pereira PN, Masatoshi N, Tagami J, Miura H (2002). Long-term durability of the dual-cure resin cement/silicon oxide ceramic bond. J Adhes Dent.

[27] Oliveira AS, Ramalho ES, Spazzin AO, Naves LZ, Moraes RR (2013). Influence of silane and solvated bonding agents on the bond strength to glass-fibre posts. Aust Endod J.

[28] Monticelli F, Grandini S, Goracci C, Ferrari M (2003). Clinical behavior of translucent-fiber posts: a 2-year prospective study. Int J Prosthodont.

